# *LincRNA00494* Suppresses Non-small Cell Lung Cancer Cell Proliferation by Regulating SRCIN1 Expression as a ceRNA

**DOI:** 10.3389/fonc.2020.00079

**Published:** 2020-02-06

**Authors:** Jingsi Dong, Bingjie Li, Dan Lin, Dan Lu, Chang Liu, Xingbing Lu, Xiaojun Tang, Lu Li, Daxing Zhu, Jiewei Liu, Xiaoming Qiu, Long Tian, Qinghua Zhou

**Affiliations:** ^1^Department of Lung Cancer Center, West China Hospital, Sichuan University, Chengdu, China; ^2^Department of Otorhinolaryngology, Head & Neck Surgery, West China Hospital, Sichuan University, Chengdu, China; ^3^Intensive Care Unit, West China Hospital, Sichuan University, Chengdu, China; ^4^Department of Laboratory Medicine, West China Hospital, Sichuan University, Chengdu, China

**Keywords:** *LincRNA00494*, ceRNA, non-small cell lung cancer, SRCIN1, LncRNA

## Abstract

**Background:** Lung cancer is the most common malignant tumor worldwide. Accumulating results have shown that long non-coding RNAs (lncRNAs) play a key role in tumorigenesis.

**Patients and Methods:** A total of 163 tumor tissues were collected from non-small cell lung cancer (NSCLC) patients from West China Hospital of Sichuan University. *LincRNA00494* is a novel lncRNA, and its expression and biological effect in NSCLC were reported in this study. NSCLC cell lines were used in this study.

**Results:** *LincRNA00494* is mainly distributed in the cytoplasm. *LincRNA00494* was downregulated in the tumor tissues compared with the adjacent non-tumor tissues. *LincRNA00494* expression was positively correlated with SRCIN1 expression (*R* = 0.57, *P* < 0.05). Silencing of *LincRNA00494* in the cell lines substantially decreased SRCIN1 expression at the mRNA and protein levels, whereas overexpression of *LincRNA00494* enhanced the SRCIN1 levels. miR-150-3p significantly decreased the luciferase signals of *LincRNA00494* and SRCIN1 reporters. After transfection with miR-150-3p mimics and miR-150-3p inhibitor, overexpression of *LincRNA00494* decreased the proliferation of the H358 (36%) and H1299 (29%) cell lines compared with that of the control cells, as shown by CCK-8 assays, whereas silencing *LincRNA00494* promoted the proliferation of the H358 (47%) and H1299 (35%) cells. Tumor growth from LincRNA00494-overexpressing xenografts was significantly decreased; additionally, LincRNA00494 silencing substantially increased tumor growth compared with that of the control cells.

**Conclusions:** Functional experiments revealed that *LincRNA00494* inhibited NSCLC cell proliferation, which might be related to the suppression of SRCIN1, a tumor suppressor gene, by acting as a decoy for miR-150-3p. The data showed that *LincRNA00494* might have antineoplastic effects during NSCLC tumorigenesis through its role as a ceRNA.

## Introduction

Lung cancer is the leading cause of cancer-related deaths worldwide (1). Due to the difficulty in early diagnosis and the poor chemotherapy response, the 5-years survival rate of lung cancer has remained at ~15% (2). Identification of for new biomarkers for the occurrence and development of non-small cell lung cancer (NSCLC) is urgently needed. Although the functions of protein-coding genes in the development of lung cancer have been extensively studied in recent decades, protein-coding genes comprise <2% of the human genome. Approximately 85% of human genomic sequences are transcribed into non-coding RNAs that are categorized into new and poorly understood RNA families (3, 4). Recent studies have shown the relationship between long non-coding RNAs (lncRNAs) and cancer subtypes, such as esophageal squamous cell carcinoma (ESCC), colorectal cancer (CRC), gastric cancer (GC), and NSCLC (5). In addition, lncRNAs may play an important role in cancer development by modulating various biological processes, including chromatin remodeling, transcription and post-transcriptional regulation. However, little is known about the specific mechanisms of lncRNAs in NSCLC.

*LincRNA00494* (located at 20q13.13: 48359911…48370638, 10.1 kb) is a novel long intergenic non-protein coding gene, and its function has not been fully elucidated. LincRNA00494 showed low expression in esophageal cancer in a previous screen (6). Furthermore, we independently verified LincRNA00494 in NSCLC. LincRNA00494 was also found to be poorly expressed in NSCLC tissues. In the present study, we demonstrated that *LincRNA00494* was downregulated in NSCLC tissues compared with the corresponding adjacent non-tumor tissues.

SRCIN1, a tumor suppressor gene, was reported to be inhibited by multiple microRNAs (miRNAs). MiRNA150 had a significant effect on SRCIN1 (7). LincRNAs can act by binding miRNAs. The aim of this study was to determine whether there is a targeted binding relationship between LincRNA00494 and miRNA150. Furthermore, a mechanistic investigation revealed that *LincRNA00494* might suppress NSCLC cell proliferation by decoying miR-150-3p, which targets SRCIN1, a tumor suppressor in the progression of cancers (8, 9). Our findings might reveal the underlying mechanism by which aberrant *LincRNA00494* expression promotes NSCLC tumorigenesis.

## Patients and Methods

### Study Subjects

A total of 163 tumor and adjacent tissue samples were collected from patients with NSCLC at the West China Hospital of Sichuan University. After recruitment, every participant underwent an interview involving questionnaires, and each patient provided informed consent. The study protocols were approved by the Medical Ethics Committee of Sichuan University. The clinical characteristics of all the patients are listed in Table 1.

**Table 1:** Baseline demographic and clinical characteristics of the study population.

**Characteristics**	**NSCLC**	
	***N***	**%**
Age (years)		
<40	25	15.3
40–60	85	52.1
>60	53	32.5
Sex		
Male	64	39.3
Female	99	60.7
Family history of cancer		
Yes	74	45.4
No	89	54.6
Smoking		
Never	114	69.9
Ever	49	30.1
Pathology of nsclc		
Adenocarcinoma	117	71.8
Squamous	46	28.2
Differentiation		
Good	47	28.8
Intermediate	85	52.1
Poor	31	19.0
Stage		
I	42	25.8
II	69	42.3
III	52	31.9

### Cell Culture

The NSCLC cell lines H358, HCC827, and H1299 were purchased from the Cell Bank of the Chinese Academy of Sciences (Shanghai, China). Cell culture procedures were performed as previously described (10). Briefly, the cell lines were cultured in RPMI-1640 medium supplemented with 10% fetal bovine serum, penicillin and streptomycin in a humidified 5% CO_2_ atmosphere at 37°C.

### Northern Blot Analysis and RNA *in situ* Hybridization Assay of Tumor Cells

In this study, we performed northern blotting to confirm the size of LincRNA00494. LincRNA00494 and vimentin gene expression in tumor cells was detected by RNA *in situ* hybridization using CanPatrolTM (SurExam Biotech, Guangzhou, China).

### PCR and siRNA Knockdown

RNA from the cells and tissues was isolated using TRIzol reagent. All protocols were based on the manufacturer's instructions. An ABI Prism 7500 sequence detection system (Applied Biosystems, USA) was used to test the level of *LincRNA00494*. GAPDH was used as an internal standard control. In this study, all PCR assays were performed in triplicate (11). The LincRNA00494 primers for qPCR were as follows: GGTCTGGTGTGGAGACAGTG and AGCTTGCAGCCAAGAAAAGC (reverse). Mature miR-150 and SRCIN1 expression were detected by a quantitative real-time PCR assay with miR-150-specific and SRCIN1 primers and a TaqMan probe, as previously reported (7). We applied specialized kits (Sengbio, Inc., Beijing, China) to perform siRNA knockdown of *LincRNA00494*.

### Reporter Plasmid Construction

The method for reporter plasmid construction was described in a previous study (12). psiCHECK2 (Clontech) was used to construct the plasmids psiCHECK2-LincRNA00494 (the plasmid containing *LincRNA00494*) and psiCHECK2-SRCIN1-3′UTR. DNA sequencing was used to verify the constructs.

### Transfection and Luciferase Assay

Lipofectamine 2000 (Invitrogen, CA, USA) was used to transfect the H358 and H1299 cell lines with reporter plasmids. All procedures were based on the manufacturer's instructions. As described previously, with minor modifications, the Dual-Luciferase Reporter assay system (Promega, Madison, WI, USA) was used to measure luciferase activity (13). We carried out two independent experiments, and each group included three replicates.

### Actinomycin D Assay

We also used Lipofectamine 2000 (Invitrogen) to transfect the H358 and H1299 cell lines. Moreover, the cell lines were co-transfected with miR-150 for 24 h and were exposed to actinomycin D (Sigma, St Louis, MO). As previously described, the stable expression of *LincRNA00494* was analyzed using qRT-PCR (10).

### Western Blot

Consistent with previous experimental procedures, Western blot analysis was conducted to assess SRCIN1 expression (10). Protein was extracted from the cell lines, and the immunoprecipitation samples were prepared using detergent-containing lysis buffer. Total protein (60 μg) was subjected to sodium dodecyl sulfate polyacrylamide gel electrophoresis (SDS-PAGE) and transferred to polyvinylidene difluoride (PVDF) membranes (Millipore). The membranes were incubated with primary antibodies against SRCIN1 (Cell Signaling Technology, dilution: 1:1,000) and β-actin (Proteintech, dilution: 1:1,000) overnight at 4°C, and the proteins were detected with a Phototope horseradish peroxidase Western blot detection kit (Thermo Fisher).

### Cell Viability Analysis

We used the Cell Counting Kit-8 system (Dojindo Laboratory, Kumamoto, Japan) to determine the cell viability, and all procedures were performed according to the manufacturer's instructions (13). There were six replicates for each group, and all experiments were repeated at least three times.

### Xenograft Growth of the NSCLC Cells in Nude Mice

Five-weeks-old female BALB/c nude mice were injected subcutaneously with 0.1 ml of cell suspension (with 1 × 10^6^ cells) containing H358 and H1299 control cells, *LincRNA00494*-silenced cells or *LincRNA00494*-overexpressing cells into the back flank. The tumors were measured every 2 days, and their volumes were calculated according to the following formula: volume = length × width^2^ × 0.5. This study was carried out in accordance with the principles of the Basel Declaration and the guidelines of the Institutional Animal Care and Use Committee of Sichuan University. The protocol was approved by the Institutional Animal Care and Use Committee of Sichuan University.

### Statistical Analysis

Analysis of variance and linear regression were used to detect the correlation between the expression of *LincRNA00494* and SRCIN1 in the NSCLC tissue. The differences between the two groups were assessed using paired Student's *t*-tests. A *p*-value < 0.05 was considered significant.

## Results

### Cellular Characterization of *LincRNA00494*

**Figure 1:**
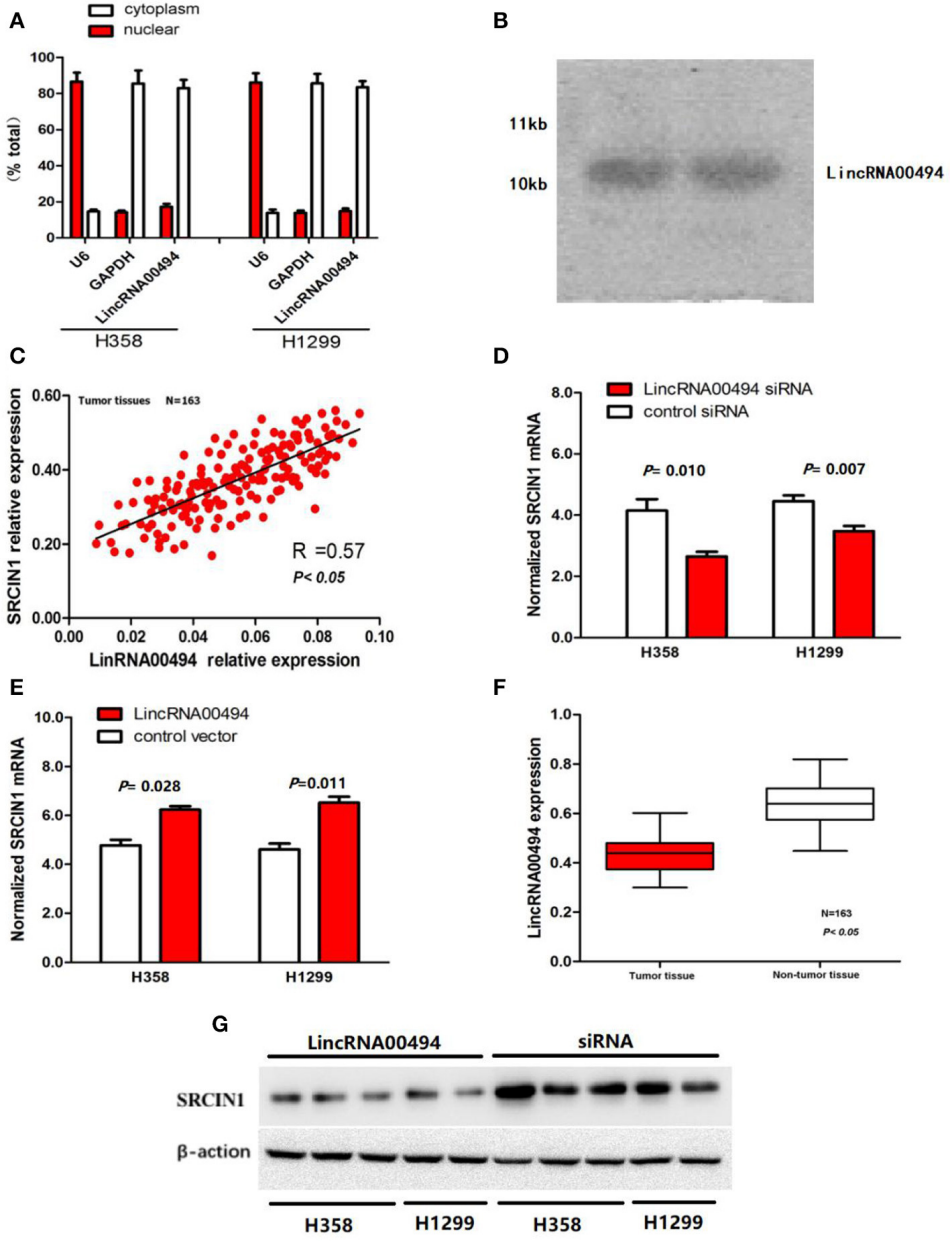
Cellular and molecular characterization of *LincRNA00494*. **(A)** The levels of the nuclear control transcript (U6), the cytoplasmic control transcript (GAPDH mRNA) and LincRNA00494 were assessed by RT-qPCR. Data are shown as the mean ± SEM. **(B)** A northern blot shows that LincRNA00494 is 10 kb. **(C)** The linear correlations between the *LincRNA00494* expression and the SRCIN1 mRNA levels were tested. The relative expression value was normalized to the GAPDH expression level. **(D,E)** *LincRNA00494* expression significantly affected SRCIN1 mRNA expression. Knockdown of *LincRNA00494* decreased the SRCIN1 expression, while ectopic expression of *LincRNA00494* increased the SRCIN1 mRNA levels. **(F)** *LincRNA00494* was expressed at a lower level in the NSCLC tissues than in the matched tumor-adjacent tissues. The expression level of *LincRNA00494* was analyzed by RT-qPCR and normalized to the GAPDH level. Data are represented as the mean ± SEM from three independent experiments. **(G)** The protein levels of SRCIN1 were assessed by Western blots of H358 cells and H1299 cells.

To determine the subcellular localization of *LincRNA00494*, we detected the mRNA levels of U6 and GAPDH via RT-qPCR. In the H358 and H1299 cell lines, RT-qPCR analysis revealed that 17.2 and 14.8% of the *LincRNA00494* transcripts were detected in the nuclear fraction, respectively, and 85.7 and 87.4% of these transcripts were found in the cytoplasmic fraction (Figure 1A). FISH shows that LincRNA00494 (red) were detected by RNA *in situ* hybridization using CanPatrolTM (Surexam Biotech, Guangzhou, China) in cytoplasm (**Figure 3A**). Meanwhile, PhyloCSF was utilized to examine the coding potential of *LincRNA00494*, and the PhyloSCF score was −149.3492, which indicated the low coding potential of *LincRNA00494*. Northern blot analysis showed that LincRNA00494 was 10 kb (Figure 1B).

### *LincRNA00494* Was Downregulated in the NSCLC Tissues

To investigate the potential key role of *LincRNA00494* in NSCLC, we detected the expression levels of *LincRNA00494* in 163 pairs of NSCLC and adjacent non-tumor tissues via RT-qPCR. The detailed clinical features are presented in Table 1. The results showed that *LincRNA00494* was strongly downregulated in the tumor tissues compared with the adjacent non-tumor tissues (Figure 1F), which suggested that *LincRNA00494* might have an antineoplastic effect during NSCLC tumorigenesis.

### Association of *LincRNA00494* and SRCIN1 in the NSCLC Tissues

According to the results mentioned above, we tested the correlation between *LincRNA00494* and SRCIN1 in the 163 NSCLC tumor tissues. The expression of *LincRNA00494* was positively correlated with the expression of SRCIN1 (*R* = 0.57, *P* < 0.05, Figure 1C). Next, we disturbed endogenous *LincRNA00494* expression by using gene overexpression and knockdown to investigate its effects on SRCIN1 expression. The results showed that when LincRNA00494 was silenced in the two cell lines, SRCIN1 was substantially decreased at the mRNA and protein levels. In contrast, the overexpression of *LincRNA00494* increased the SRCIN1 expression level (Figures 1D,E,G).

### *LincRNA00494* Regulated the SRCIN1 Expression Levels by Competing With miR-150

**Figure 2:**
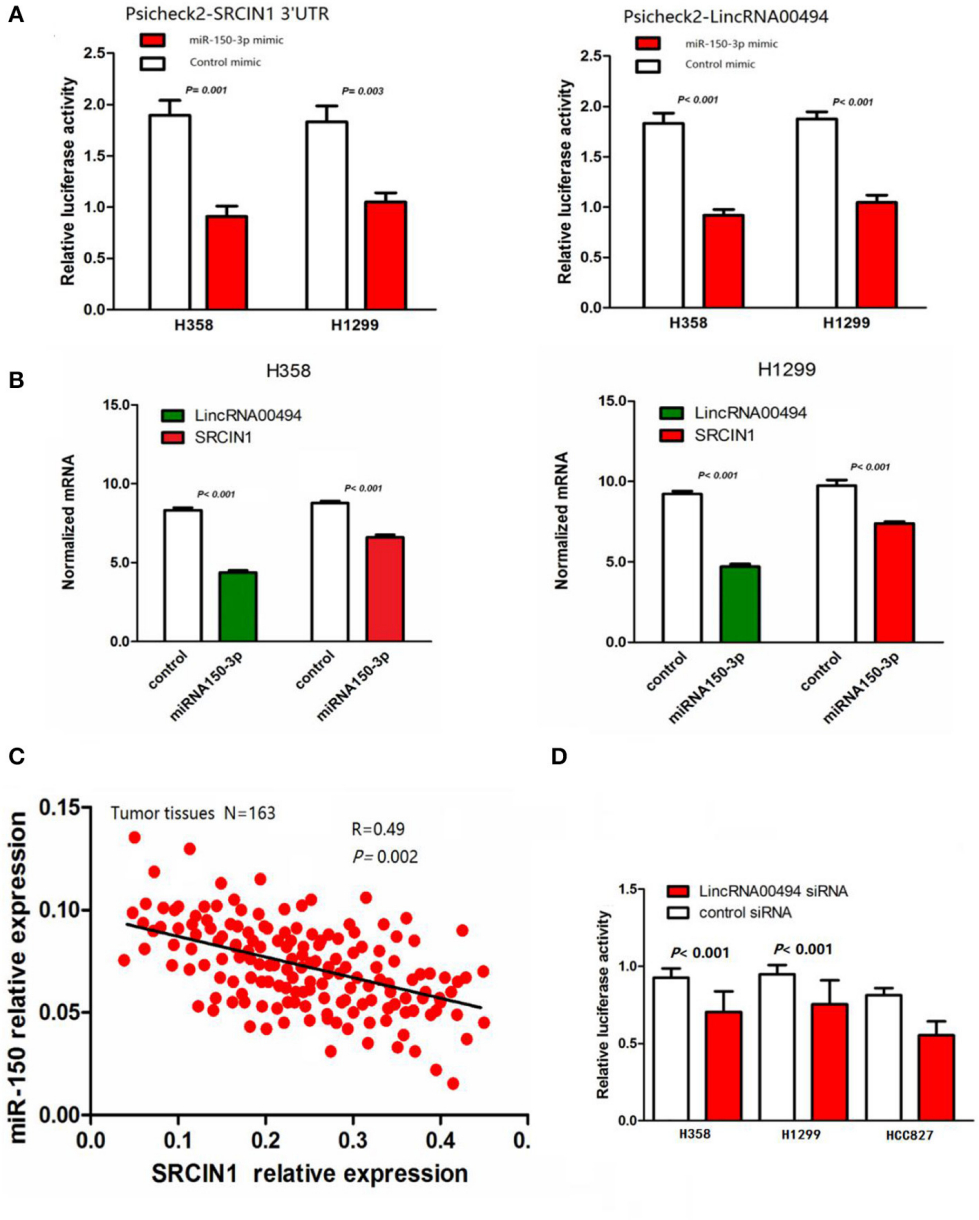
The relationships among miR-150-3p, *LincRNA00494* and SRCIN1. **(A)** Both *LincRNA00494* and SRCIN1 are targeted by miR-150-3p. miR-150-3p significantly decreased the luciferase signals of both *LincRNA00494* and SRCIN1. **(B)** The SRCIN1 and *LincRNA00494* levels were significantly decreased. Data are shown as the mean ± SEM (normalized to GAPDH). **(C)** The negative correlations between the *LincRNA00494* expression levels and the SRCIN1 mRNA levels were tested. **(D)** The reporter vector was cotransfected into the NSCLC cells with LincRNA00494 siRNA or control siRNA. The luciferase signal was substantially decreased.

miR-150-3p was predicted to be the target of both *LincRNA00494* and SRCIN1. We cloned the 3′ untranslated region (UTR) of SRCIN1 and *LincRNA00494* into the psiCHECK2 vector and cotransfected these reporters with miR-150-3p mimics in the NSCLC cells to verify the role of miR-150-3p in the two NSCLC cell lines. miR-150-3p notably decreased the luciferase activity of SRCIN1 and *LincRNA00494*, as shown in Figure 2A. Moreover, we measured the mRNA levels of SRCIN1 and *LincRNA00494* in the NSCLC cells after treatment with miR-150-3p mimics. As shown in Figure 2B, the SRCIN1 and *LincRNA00494* levels were notably decreased. Furthermore, the expression levels of SRCIN1 and miR-150-3p in the tumor tissues of the 163 NSCLC patients were detected, and we identified a negative relationship between the miR-150-3p and SRCIN1 levels (*R* = 0.49, *P* = 0.002; Figure 2C). Subsequently, we cotransfected psiCHECK2-SRCIN1 3′UTR with *LincRNA00494* siRNA. The results demonstrated that the knockdown of *LincRNA00494* had a negative effect on the luciferase intensity in the H358, H1299, and HCC827 cells (Figure 2D). All these results showed that miR-150-3p could target both *LincRNA00494* and SRCIN1. Thus, miR-150-3p, as a molecular decoy, regulates SRCIN1 expression via LincRNA00494.

### *LincRNA00494* Modulated Tumor Cell Growth

**Figure 3:**
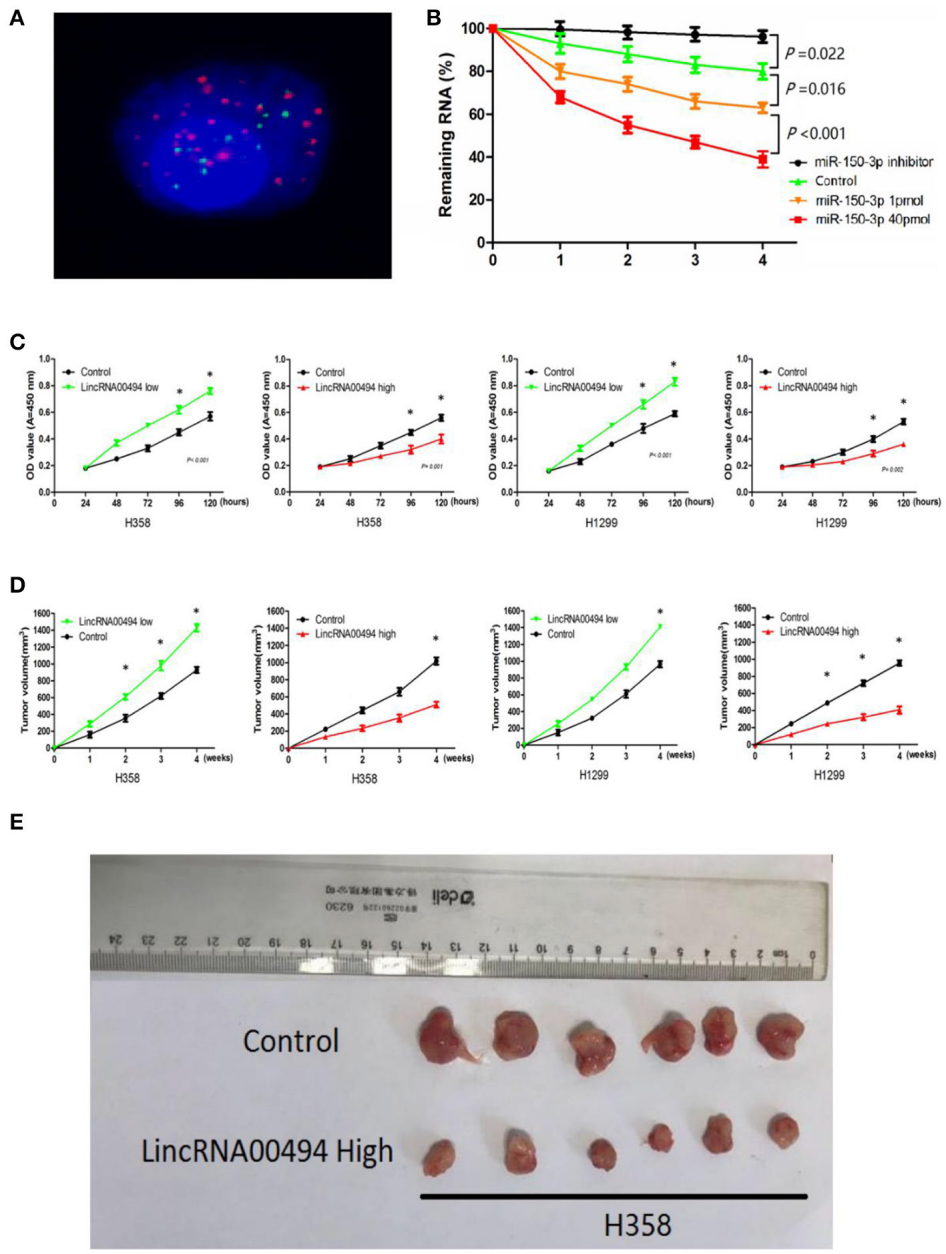
*LincRNA00494* mediated proliferation of the NSCLC cells. **(A)** FISH showed that LincRNA00494 was mostly located in the cytoplasm, with a small portion in the nucleus. Green indicates vimentin; red indicates LincRNA00494. **(B)** Cells were harvested, and the RNA stability of *LincRNA00494* was analyzed by RT-qPCR relative to time 0 after new RNA synthesis was blocked with actinomycin D; the data are shown as the mean ± SEM (normalized to GAPDH). **(C)** H358 and H1299 cells were seeded in 96-well plates after transfection, and the cell proliferation was assessed daily for 5 days using the CCK-8 assay. Six replicates were performed for each group, and the experiment was repeated three times. Data are shown as the mean ± SEM. **(D,E)** The data show the tumor volumes of the xenografts in each group 4 weeks after subcutaneously implantation of stable NSCLC cells. The mean tumor volumes from six nude mice from each group are shown at different time points.

Next, we investigated the effect of miR-150-3p on the RNA stability of *LincRNA00494*. We transfected miR-150-3p mimics and miR-150-3p inhibitor into the cells, and *LincRNA00494* was downregulated due to the inhibition of RNA synthesis by actinomycin D in the presence of miR-150-3p (Figure 3B). As shown in Figure 3C, the proliferation of the H358 (36%) and H1299 (29%) cell lines decreased after overexpression of *LincRNA00494*. Silencing *LincRNA00494* promoted the proliferation of the H358 (47%) and H1299 (35%) cells.

### *LincRNA00494* Accelerated the Tumor Growth of the Xenografts

For confirmation of the importance of *LincRNA00494* in tumor growth, we performed subcutaneous injection of NSCLC cells for the generation of xenografts. As shown in Figures 3D,E, the overexpression of *LincRNA00494* decreased the growth of xenografts; additionally, *LincRNA00494* silencing substantially increased the tumor growth compared with that of the control cells.

## Discussion

In the present study, we examined *LincRNA00494* in NSCLC and showed that it was dramatically downregulated, indicating the potential antineoplastic function of *LincRNA00494* in NSCLC. Our work illustrated the associations among *LincRNA00494*, miR-150-3p and SRCIN1. Our findings demonstrated the important anticarcinogenic role of *LincRNA00494* via decoying miR-150-3p, which could target SRCIN1.

LncRNAs are becoming important factors in various basic biological processes, and an increasing number of studies have proposed that lncRNAs have critical roles in cancer development (14, 15). Numerous lncRNAs have been implicated in lung cancer; however, only a few of these molecules have been characterized and shown to have specific biological functions and potential mechanisms. HOTAIR is a well-known oncogenic lncRNA that is highly expressed in NSCLC, SCLC, and various other human cancers (16). The expression of MALAT1 is positively correlated with the proliferation and metastasis of tumor cells (17). Moreover, the expression of CCAT2 was upregulated in NSCLC tissues compared to paired adjacent normal lung tissues. CCAT2 upregulation was reported in lung adenocarcinoma but not in squamous cell carcinoma (17). LncRNAs can be categorized into oncogenic LncRNAs and tumor suppressor LncRNAs according to their deregulated expression in cancer cells, similar to protein-coding genes. MEG3 is believed to be a tumor suppressor lncRNA because its expression is decreased in various human tumors, including lung cancer tissues (18). TUG1 was significantly downregulated in lung cancer tissues compared with the corresponding normal lung tissues. Downregulation of TUG1 was also positively related to advanced pathological stage, increased tumor size and decreased survival time in both lung squamous cell carcinoma and adenocarcinoma (19). Similarly, SPRY4-IT1 downregulation promoted the migration and invasion of A549 cells *in vitro*, whereas SPRY4-IT1 overexpression facilitated apoptosis (20). In our study, *LincRNA00494* was significantly downregulated in the NSCLC tissues compared to the corresponding non-tumor lung tissues.

Next, we investigated the antineoplastic mechanism of LincRNA00494. Our data showed that the proliferative capacity of NSCLC cells was accelerated after *LincRNA00494* silencing. More importantly, our results also suggested that *LincRNA00494* acts as a molecular decoy for miR-150-3p. miRNAs are short RNA sequences that negatively regulate gene expression by targeting the 3′UTRs of mRNAs. miRNAs mediate many biological functions in tumors, such as cell proliferation, differentiation, and migration. Furthermore, miRNAs play an important role in gene regulation by targeting many coding and non-coding genes. Numerous reports have suggested that miRNAs mediated their effects by targeting lncRNAs (21). Our experiments demonstrated that lncRNAs may have an effect on their targets by acting as decoys for miRNAs, which is a very important mechanism.

Our work found that SRCIN1 served as a critical component of the *LincRNA00494*-miRNA network. The protein p140CAP, an important member of the Cap family encoded by SRCIN1, is expressed in various human tissues, and the expression level of p140CAP in tumors is low (8, 22). The silencing of p140CAP, a tumor suppressor gene, promoted tumor cell growth independent of anchoring and enhanced tumor growth and development (8, 23). The Cap protein p140CAP can inhibit the downstream signaling pathway of Src and regulate the activity of focal adhesion kinase and Ras/extracellular signal-related kinases and thus has an anticancer role. Src is a tyrosine kinase that is often overexpressed or abnormally activated in cancer cells. Mounting evidence has demonstrated that Src activity is elevated in lung cancer cells. SRCIN1, a newly identified inhibitor of Src, is the only gene that is negatively regulated by miR-150-3p (7). Through luciferase reporter analysis, we demonstrated that SRCIN1 was repressed by miR-150-3p, and this function was suppressed by the overexpression of *LincRNA00494*. Furthermore, we found that SRCIN1 expression was gradually improved, along with increased levels of *LincRNA00494*.

## Conclusion

Collectively, our results suggest that LincRNA00494 may enhance SRCIN1 expression by competing with miR-150-3p, thereby mediating NSCLC cell proliferation.

## Data Availability Statement

All datasets for this study are included in the article/supplementary material.

## Ethics Statement

The studies involving human participants were reviewed and approved by Medical Ethics Committee of Sichuan University. The patients/participants provided their written informed consent to participate in this study.

## Author Contributions

JD and QZ conceived and designed the experiments. JD, BL, and JL performed the experiments. CL, LT, and DLu analyzed the data. LL, XL, DZ, and XT provided the reagents, materials and analysis tools. JD, DLi, and XQ contributed to the manuscript preparation.

### Conflict of Interest

The authors declare that the research was conducted in the absence of any commercial or financial relationships that could be construed as a potential conflict of interest.
